# Modeling the Transmission of Middle East Respirator Syndrome Corona Virus in the Republic of Korea

**DOI:** 10.1371/journal.pone.0144778

**Published:** 2015-12-21

**Authors:** Zhi-Qiang Xia, Juan Zhang, Ya-Kui Xue, Gui-Quan Sun, Zhen Jin

**Affiliations:** 1 Department of Mathematics, North University of China, Taiyuan, Shanxi 030051, PR China; 2 Complex Systems Research Center, Shanxi University, Taiyuan, Shanxi 030006, PR China; Kyushu University, JAPAN

## Abstract

The 2015 epidemic of Middle East respiratory syndrome (MERS) in the Republic of Korea has been the largest outbreak outside Middle East. This epidemic had caused 185 laboratory-confirmed cases and 36 deaths in the Republic of Korea until September 2, 2015, which attracted public’s attention. Based on the detailed data of patients released by World Health Organization (WHO) and actual propagation of the epidemic, we construct two dynamical models to simulate the propagation processes from May 20 to June 8 and from June 9 to July 10, 2015, respectively and find that the basic reproduction number *R*_0_ reaches up to 4.422. The numerical analysis shows that the reasons of the outbreak spread quickly are lack of self-protection sense and targeted control measures. Through partial correction analysis, the parameters *β*_1_ and *γ* have strong correlations with *R*_0_, i.e., the infectivity and proportion of the asymptomatic infected cases have much influence on the spread of disease. By sensitivity analysis, strengthening self-protection ability of susceptible and quickly isolating or monitoring close contacts are effective measures to control the disease.

## Introduction

The 2015 epidemic of Middle East respiratory syndrome (MERS) in the Republic of Korea has been the largest outbreak outside Middle East since the first case was identified in Jeddah, Saudi Arabia, in June 2012 [1–4]. A total of 1,413 laboratory-confirmed cases infected by MERS-CoV, including 502 deaths, had been reported globally as of August 19, 2015 [5], and mortality rate was about 35% [6]. Most cases emerged in the Middle East, sporadic cases had been reported in UK, Italy, France, Spain, United States and so on [7–10]. Most of these cases were confirmed after a traveling in Middle East. In the Republic of Korea, the first case was an import one having a traveling experience in Middle East [8, 11]. Due to poor pre-control measures, the number of additional confirmed cases gradually increased during the period from May 20, 2015 to June 8, 2015. Then the Korean Government took effective control measures and the number of additional confirmed cases gradually decreased during the period from June 9 to July 4 [12, 13]. Therefore, the Korean Government and WHO announced the end of MERS epidemic in the Republic of Korea in the middle of July.

It is necessary to learn about the source of infection and transmission mechanism of MERS. The most probable source of Middle East respiratory syndrome coronavirus (MERS-CoV) is bats, and MERS-CoV-related viruses have been detected in bats. However, people have little chance to contact with them directly. It is generally considered that dromedary camels are the intermediate reservoirs for MERS-CoV transmission from bats to human, but the accurate role of camels in transmission of MERS-CoV has not been available [1, 10, 14, 15]. Generally, there are two main transmission routes [4, 8, 16–18]: one is animal-to-human transmission, the other one is human-to-human transmission. The MERS-CoV can cause diseases ranging from the common cold to Severe Acute Respiratory Syndrome (SARS). The patients infected by MERS-CoV will have symptoms including fever, cough and shortness of breath, and the severe patients may have renal failure [1, 6, 7, 14, 19]. However, humans do not immediately show clinical symptoms after being infected and they undergo a incubation period from 2 to 14 days, during which time they are not infectious [11, 16].

Why the MERS broke out in the Republic of Korea rather than other countries such as UK, France, Italy is worth researching. It is necessary to have a look at how the epidemic emerged and developed in the Republic of Korea [8, 20, 21]. The first laboratory-confirmed patient was reported on May 20, 2015. He is a 68 year-old male who had traveled in several countries in Middle East before flying back to the Republic of Korea on May 4. Until May 11 he showed symptoms and sought for three hospitals before he was finally confirmed and isolated. During the period from being infected to being confirmed he had a close contact with many visitors, relatives, inpatients and health care workers. The new cases continued to be reported in the next few days, and they all had corrections with the first confirmed case. Through analyzing the actual transmission process, several factors appear to have contributed to the initial spread of this virus [22]:

1. The appearance of MERS-CoV was unexpected and unfamiliar to most physicians;
2. The practise of seeking hospitals may be a contribution factor;
3. The control measures of the hospitals are not optimal;
4. Multi-beds rooms in the hospitals are a contribution factor;
5. The custom of many friends and relatives to visit the patient is also a contribution factor.

A lot of work has been done in studying MERS. Lee et al [8] characterized the transmission chains of MERS-CoV infection in South Korean’s outbreak. Nishura et al [21] estimated the expected size of MERS clusters and the number of generations using a stochastic epidemic model. Chowell et al [16] studied a MERS-CoV transmission model with index and secondary cases. Rivers et al [23] divided the susceptible populations into the high-risk group *S*_1_ and the normal or low risk group *S*_2_.

In order to describe the propagation process of MERS in the Republic of Korea during this period, we propose a deterministic SEAIHR model to study the spread of MERS among humans. Because there is no zoonotic infections of MERS-CoV in the Republic of Korea, only one transmission route (human-to-human infection) is considered in model. Since the disease is almost distributed throughout the Republic of Korea, all the Korean are viewed as research subject. Based on infection status, the population is divided into the following categories: susceptible individual, exposed individual, asymptomatic individual, symptomatic infected individual, hospitalized case and removed case [16]. According to the practical situation, there is no effective control measures included in our model before June 8, 2015.

## Materials and Methods

### Data

Time series of reported cases were reported by the WHO [24] and [32]. They provide detailed data of the epidemic in the Republic of Korea, including patient’s age, gender, data of symptoms, data of first hospitalization, data of laboratory confirmation and so on. We can make a related analysis of MERS based on the nice data.

### Propagation Mechanism

The spread process (Table 1) of the disease is considered as follows: when a susceptible individual is infected, he or she turns to be an exposed case. Few days later, parts of the exposed cases turn to be asymptomatic cases (although no symptom, they have the ability to infect others), the remainings turn to be symptomatic cases. The symptomatic cases will go to hospital for treatment soon. After treatment, some of them will recover, and about 21% will die [25]. The asymptomatic cases will self-recover without any treatment.

**Table 1:** Transmission rules of compartmental Model (1).

Number	Transition	Transition rate
(1)	(*S*, *E*) → (*S* − 1, *E* + 1)	$\beta_{1}\frac{SA}{N}+\beta_{2}\frac{SI}{N}+\beta_{3}\frac{SH}{N}$
(2)	(*E*, *A*) → (*E* − 1, *A* + 1)	(1 − *γ*)*σE*
(3)	(*E*, *I*) → (*E* − 1, *I* + 1)	*γσE*
(4)	(*A*, *R*) → (*A* − 1, *R* + 1)	*k*_1_ *A*
(5)	(*I*, *H*) → (*I* − 1, *H* + 1)	*λI*
(6)	(*H*, *R*) → (*H* − 1, *R* + 1)	*k*_2_ *A*

### Dynamical model without effective control measures

In order to facilitate research and make our model more reasonable, some assumptions are made:

1. The whole population is initially susceptible except the first confirmed case;
2. There is no zoonotic infections of MERS-CoV in the Republic of Korea, only considering the epidemic spread in the human beings;
3. There is no effective control measures before June 8, 2015.

**Fig 1:**
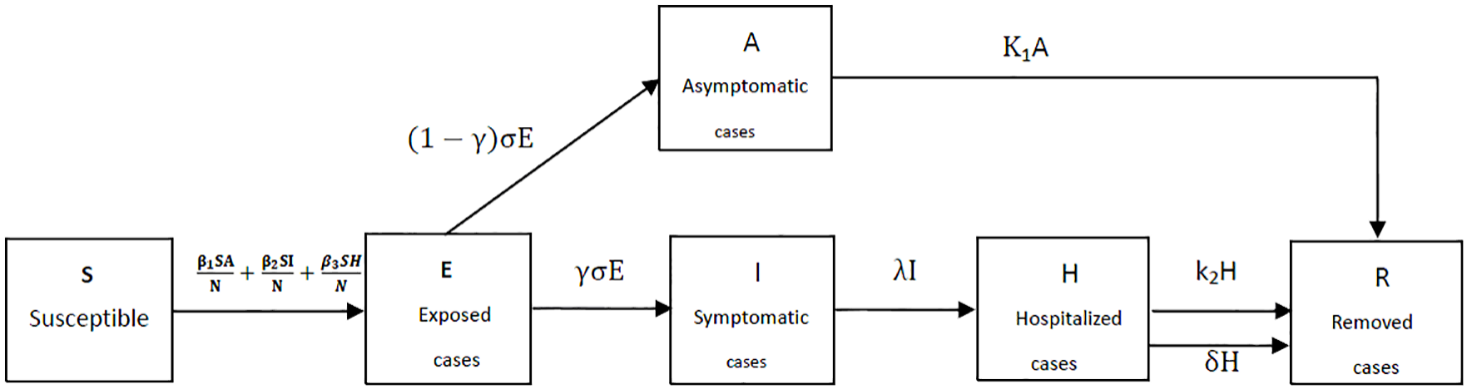
Flow diagram of the compartmental model of MERS in the Republic of Korea.

Based on the above assumptions, the spread of MERS in the human begins is shown in Fig 1. And the corresponding dynamical model is given in Eq (1):

$$\left\{\begin{array}{c} \frac{dS}{dt}=-\beta_{1}\frac{SA}{N}-\beta_{2}\frac{SI}{N}-\beta_{3}\frac{SH}{N},\\ \frac{dE}{dt}=\beta_{1}\frac{SA}{N}+\beta_{2}\frac{SI}{N}+\beta_{3}\frac{SH}{N}-\sigma E,\\ \frac{dA}{dt}=\left(1-\gamma \right)\sigma E-k_{1}A,\\ \frac{dI}{dt}=\gamma \sigma E-\lambda I,\\ \frac{dH}{dt}=\lambda I-k_{2}H-\delta H,\\ \frac{dR}{dt}=k_{1}A+k_{2}H+\delta H, \end{array}\right.$$ (1)

where *S* denotes the number of susceptible individuals; *E*, the number of exposed individuals; *A*, the number of asymptomatic infected cases; *I*, the total number of mild infected person and severe patients. *H*, the number of hospitalized cases; *R*, the number of removed cases. *N*, the total number of human population in the Republic of Korea.

*β*_1_ is the transmission coefficient of the asymptomatic infected cases, *β*_2_ is the transmission coefficient of the symptomatic infected cases (mild infected person and severe patients) to the susceptible, *β*_3_ is the transmission coefficient of the hospitalized cases to the susceptible, $\frac{1}{\sigma }$ is the mean time of incubation period, $\frac{1}{\lambda }$ is the mean time from data of symptoms onset to data of hospitalization, $\frac{1}{k_{1}}$ is the mean infectious period of asymptomatic infected person for survivors, $\frac{1}{k_{2}}$ is the mean duration for hospitalized cases for survivors, $\frac{1}{\delta }$ is the mean duration from hospitalization to death, *γ* is clinical outbreak rate in all the infected cases. The unit in Model (1) is taken as *days* ^−1^.

### Estimation of basic reproduction number *R*_0_ in the Republic of Korea

The transmission coefficients *β*_1_, *β*_2_, *β*_3_ and *γ* are unknown. On the basis of the actual reported confirmed cases, we use the least-squares method to estimate these parameter values such that the sum of squared errors between the actual data and the solution of Eq (2) is minimum [28–30]. The accumulated confirmed incidence (*I*_*total*_) in the Republic of Korea is fitted to estimate four parameters (*β*_1_, *β*_2_, *β*_3_ and *γ*):

$$\frac{dI_{total}}{dt}=\gamma \sigma E.$$ (2)

**Fig 2:**
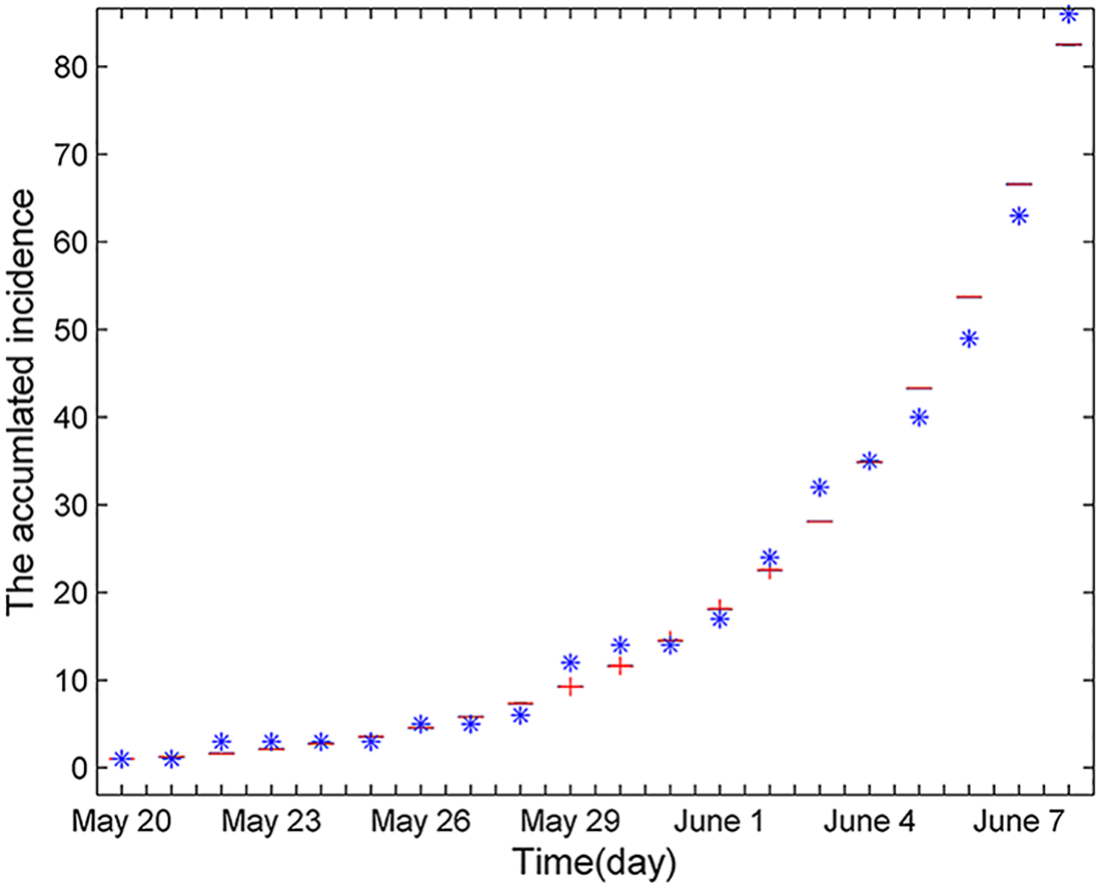
Fitting curve of real data from May 20, 2015 to June 8, 2015 by Model (1), where the blue dots denote real data obtained from [24].

Denoting the actual data of accumulated confirmed patients from May 20 to June 8, 2015 as $\bar{I_{total}}$. The estimation process is as follows (see Fig 2): first, construct a function $f=\sum_{t=1}^{n}{\left[I_{total}\left(t\right)-\bar{I_{total}}\left(t\right)\right]}^{2}$ (where *n* is the number of actual data *I*_*total*_(*t*) which is the accumulated data of the Eq (2)); second, find a optimal set of parameter values to minimize the value of *f* by applying MATLAB.

Through the least-squares method, the optimal set of parameter values is *β*_1_ = 0.8756, *β*_2_ = 0.7833, *β*_3_ = 0.4568 and *γ* = 0.0348.

Substituting the parameter values (they can be found in Table 2) into the expression of the basic reproduction number *R*_0_ (see S1 File), and estimate *R*_0_ = 4.422 > 1 in the early epidemic. It is well-known that if *R*_0_ is bigger than one, disease will spread in the human beings. This indicates that the disease spread quickly due to insufficient control measures and other reasons.

**Table 2:** Parameters value in system Eq (1).

Parameter	Range	Mean and 95%CI	Source
$\frac{1}{\sigma }$	(3∼8)	5.2	[16]
$\frac{1}{\lambda }$	(3∼7)	5	[16]
$\frac{1}{k_{1}}$	(3∼7)	5	[16]
$\frac{1}{k_{2}}$	(5∼10)	7	[16]
$\frac{1}{\delta }$	(0∼42)	15.16	Calculated
*β*_1_	-	0.8756(0.853 ∼ 0.9324)	Estimated
*β*_2_	-	0.7833(0.5925 ∼ 0.8592)	Estimated
*β*_3_	-	0.4568(0.3839 ∼ 0.6751)	Estimated
*γ*	-	0.0348(0.0285 ∼ 0.353)	Estimated
*N*	49520000	-	[26]
*S*_0_	49519960	-	[26]
*E*_0_	16 ∼ 32	-	[27]
*A*_0_	16 ∼ 36	-	[27]
*I*_0_	1	-	[27]
*H*_0_	0	-	[27]
*R*_0_	0	-	[27]

### The model with control measures in the Republic of Korea

**Fig 3:**
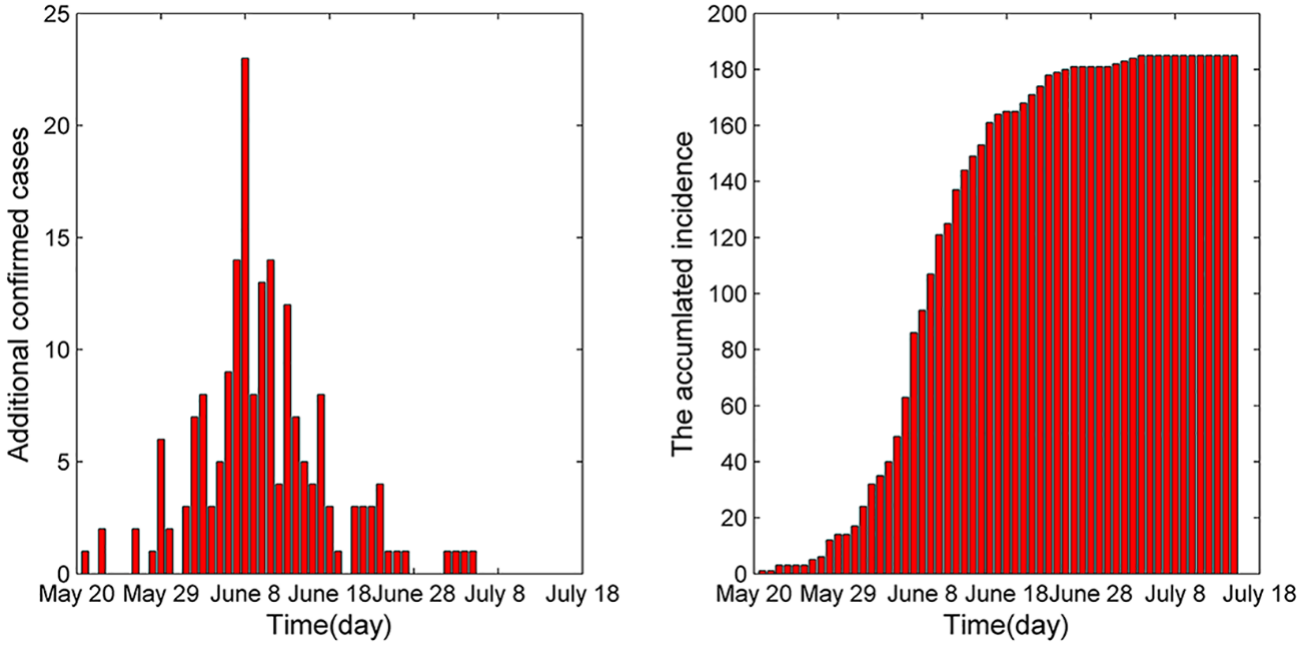
The number of confirmed cases.

The number of additional confirmed cases performs downward trend (Fig 3) from June 9, 2015. There is no additional confirmed cases from July 5, 2015, which illustrates that the control measures took by the Republic of Korea controlled the spread of the epidemic and eventually defeated the disease effectively. Here, we list the main control measures [5, 6, 22]:

1. Early and complete identification and investigation of all contacts;
2. Robust quarantine/isolation and monitoring of all contacts and suspected cases;
3. Full implementation of infection prevention and control measures;
4. Prevention of travel, especially international, of infected persons and contacts;
5. Washing your hands regularly with soap and water and maintaining good personal hygiene;
6. Avoiding close contact with people who are sick;
7. Covering your mouth and nose with a tissue or your sleeve when coughing or sneezing.

It is found that the main reasons of successfully controlling and eliminating disease can divide into two categories: ①: improving of self-protecting ability of susceptible. ②: isolating or monitoring all the close contacts, where asymptomatic cases and exposed cases are all belong to close contacts, and the confirmed cases and hospitalized cases are forcefully isolated. As of August 25, 2015, a total number of 16,693 cases were isolated or monitored [25]. Based on this situation we establish a dynamical Model (3) with control measures as follows:

$$\left\{\begin{array}{cc} & \frac{dS}{dt}=-\frac{l_{1}\beta_{1}SA}{N}-\frac{l_{2}\beta_{2}SI}{N}-\frac{l_{3}\beta_{3}SH}{N},\\ & \frac{dE}{dt}=\frac{l_{1}\beta_{1}SA}{N}+\frac{l_{2}\beta_{2}SI}{N}+\frac{l_{3}\beta_{3}SH}{N}-\sigma E-d_{1}E,\\ & \frac{dA}{dt}=\left(1-\gamma \right)\sigma E-k_{1}A-d_{2}A,\\ & \frac{dI}{dt}=\gamma \sigma E-\lambda I-d_{3}I,\\ & \frac{dH}{dt}=\lambda I-k_{2}H-\delta H-d_{4}H,\\ & \frac{dR}{dt}=k_{1}A+k_{2}H+\delta H+d_{1}E+d_{2}A+d_{3}I+d_{4}H, \end{array}\right.$$ (3)

where *d*_1_, *d*_2_, *d*_3_ and *d*_4_ are isolation or monitoring rates in *E*, *A*, *I* and *H* compartment, respectively. *l*_1_, *l*_2_ and *l*_3_ are self-protection coefficients with asymptomatic cases, symptomatic cases and hospitalized cases, respectively.

## Results

### Sensitivity and uncertainty analysis of basic reproduction number *R*_0_

The basic reproduction number is the threshold whether disease will outbreak and be prevalent in the crowd. When *R*_0_ > 1, the disease will spread in the population; when *R*_0_ < 1, the disease will gradually disappear [18, 29, 31]. So it is important to study the sensitivity and uncertainty analysis of basic reproduction number *R*_0_ on crucial parameters. Here we adopt Latin hypercube sampling (LHS) to study the correlation between five crucial parameters (*β*_1_, *β*_2_, *β*_3_, *γ*, *λ*) and *R*_0_ to asses the effect of corresponding control measures.

Randomly choosing 1000 samples of the five crucial parameters (*β*_1_, *β*_2_, *β*_3_, *γ*, *λ*), which follow a normal distribution. Based on the 1000 samples, we perform the sensitivity and uncertainty analysis through rank correlation coefficient(PRCC), there is such a relationship: the bigger the absolute value of PRCC, the stronger the correlation between the chosen parameter and *R*_0_. From Table 3, it is easy to find that the absolute value of PRCC between *β*_1_ and *R*_0_ is biggest, which shows that *β*_1_ is the most important in determining *R*_0_. It also indicates that the key factor of the rapid spread of the epidemic in the early stage is no timely isolating close contacts with the patients. The parameter *λ* has a negative correction with *R*_0_, i.e., when shorting the time from the onset to hospitalization, the value of *R*_0_ will decrease.

**Table 3:** Partial rank correlation coefficient (PRCC) for the basic reproduction number *R*_0_ and each input parameter variable.

Input parameter	PRCC
*β*_1_	0.83424
*β*_2_	0.0510
*β*_3_	0.018
*γ*	0.5377
*λ*	-0.2042

### Parameter estimation

**Fig 4:**
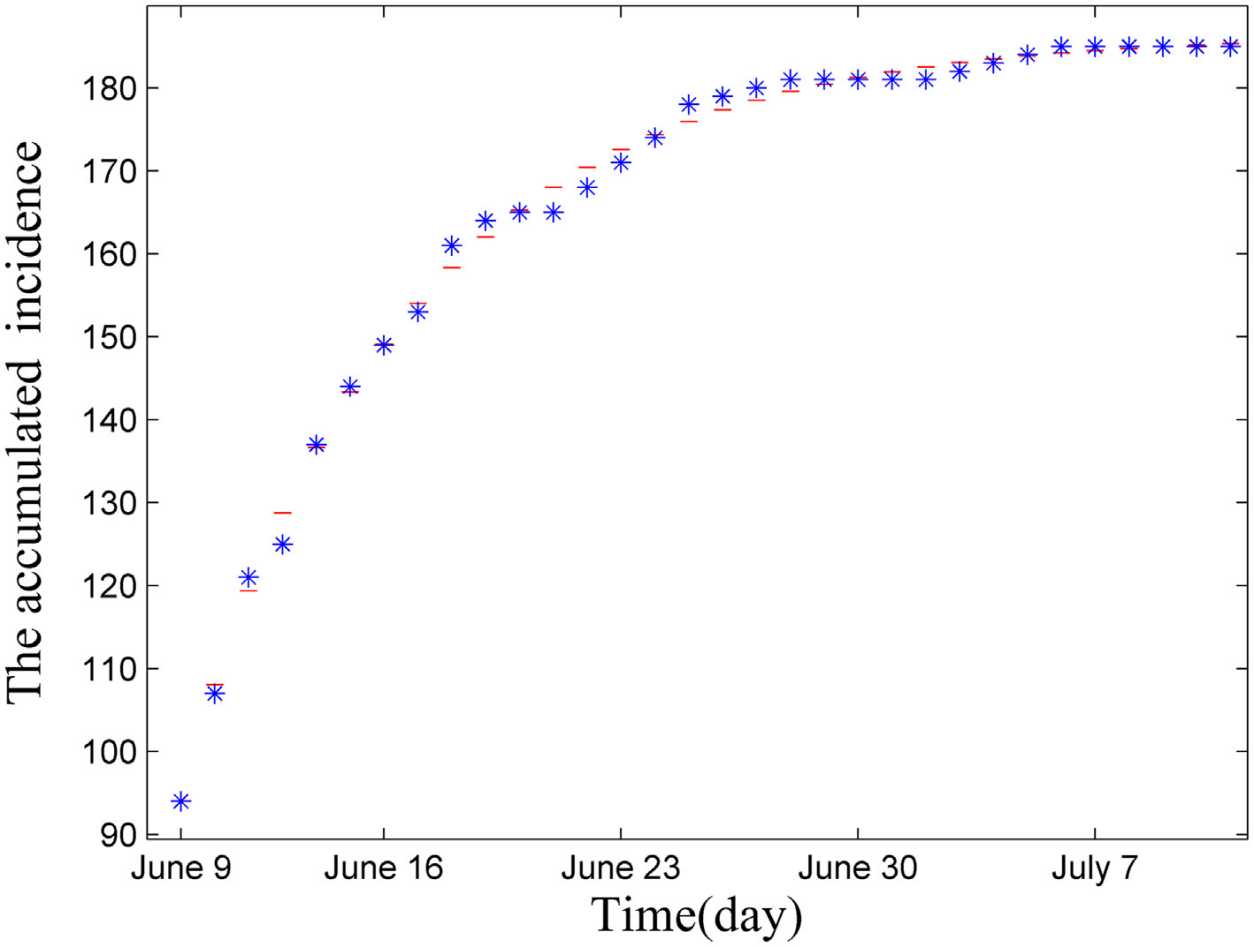
Fitting curve of real data from June 9, 2015 to July 10, 2015 by deterministic Model (3), where the blue dot denotes the real data of accumulated incidence.

In this section, as the same method in estimating *β*_1_, *β*_2_ and *β*_3_, we apply the least squares method to estimate *l*_1_, *l*_2_, *l*_3_, *d*_1_, *d*_2_, *d*_3_ and *d*_4_ (see Table 4). We assume that *d*_1_, *d*_2_, *d*_3_ and *d*_4_ are the same, and the fitting curve is shown in Fig 4.

**Table 4:** Parameter values and initial value in model Eq (3).

Parameter			Initial variable		
Parameter	Value	Source	Initial variable	Value	Source
*l*_1_	0.3239	Estimated	*S*(0)	49516000	[*A*]
*l*_2_	0.9175	Estimated	*E*(0)	2353	[*A*]
*l*_3_	0.5316	Estimated	*A*(0)	1027	[*A*]
*d*_1_	0.1922	Estimated	*I*(0)	43	[*A*]
*d*_2_	0.1922	Estimated	*H*(0)	20	[*A*]
*d*_3_	0.1922	Estimated	*R*(0)	1047	[*A*]
*d*_4_	0.1922	Estimated	$\bar{I_{total}}$	94	[27]

[*A*] denotes that the initial value (*S*(0), *E*(0), *A*(0), *I*(0), *H*(0), *R*(0)) is obtained from Model (3).

$\bar{I_{total}}$ is accumulated confirmed cases.

Substituting parameter values into the expression of *R*_*c*_ (see S1 File), and get *R*_*c*_ = 0.385 which is less than one, this also illustrates that MERS epidemic in the Republic of Korea will eventually disappear, which is consistent with the actual situation that there is no additional confirmed cases form July 5, 2015.

### Sensitivity analysis of the basic reproduction number *R*_*c*_

In this section, we apply two-dimension figures to explain how parameters affect the value of *R*_*c*_.

**Fig 5:**
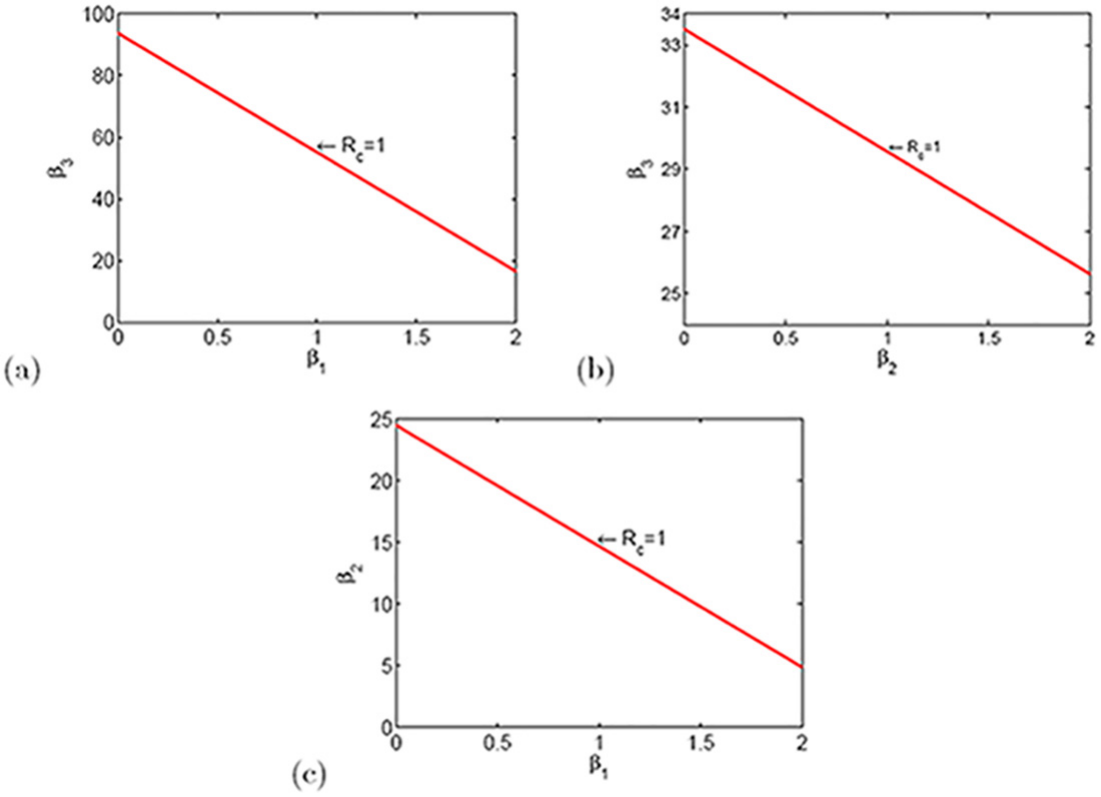
The combined influence of transmission coefficients on *R*_*c*_.

**Fig 6:**
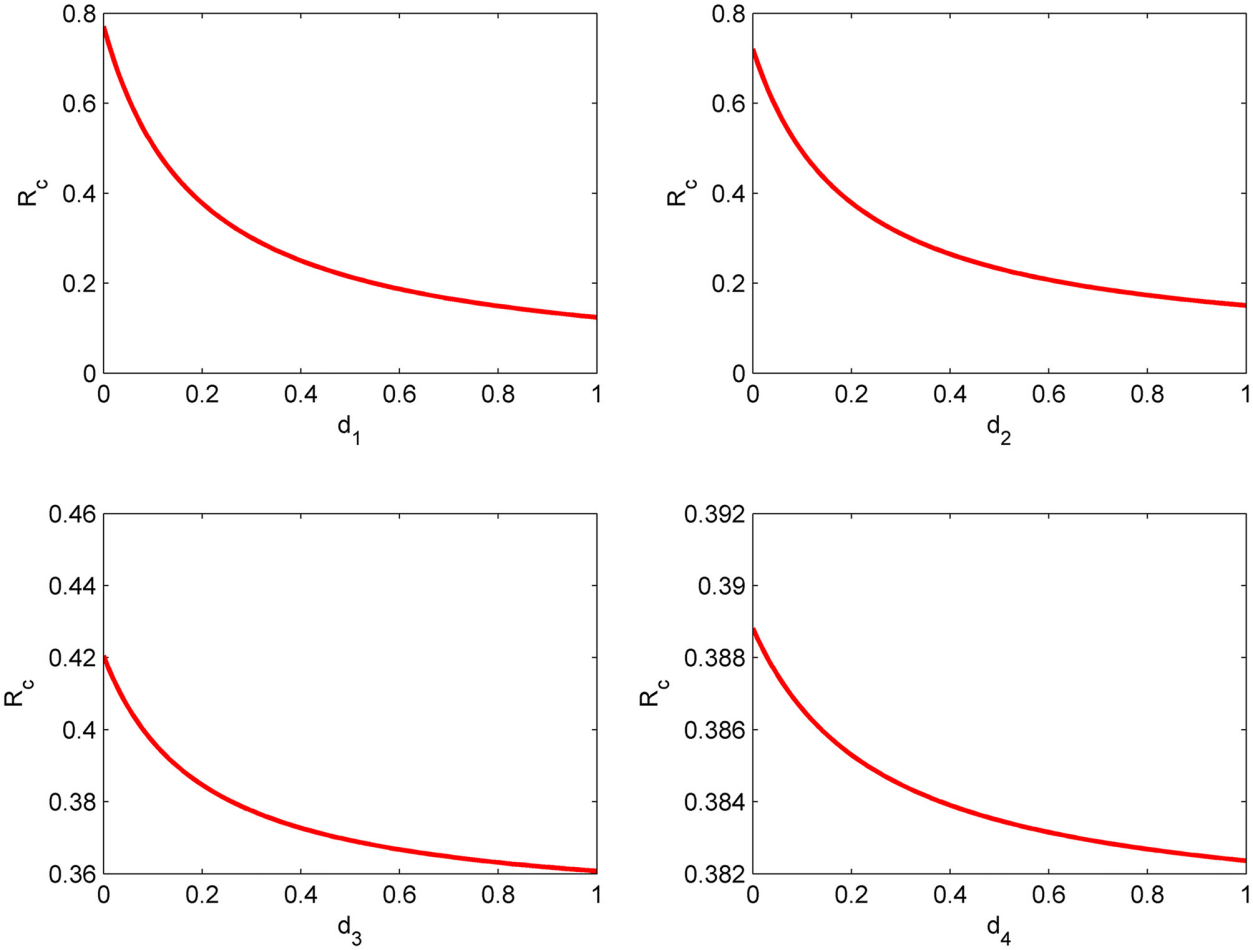
The relationships between isolation or monitor rate (*d*_1_, *d*_2_, *d*_3_ and *d*_4_) and *R*_*c*_.

From Fig 5, it is apparent that when a transmission coefficient is fixed, how the other two transmission coefficients determine the value of *R*_*c*_. The region on the left of the red line represents *R*_*c*_ < 1. The region on the right of the red line represents *R*_*c*_ > 1. In Fig 6, it shows the change of *R*_*c*_ with respect to *d*_1_, *d*_2_, *d*_3_ and *d*_4_, respectively. It is also easy to notice that when the isolating or monitoring rate increases, the basic reproduction number *R*_*c*_ will reduce, where *d*_1_ and *d*_2_ have a bigger influence on *R*_*c*_ than *d*_3_ and *d*_4_. It tells us that quickly finding and isolating exposed and asymptomatic cases can effectively control the spread of epidemic.

### The relationship between self-protective coefficient, transmission coefficient and the final size of accumulated confirmed cases

**Fig 7:**
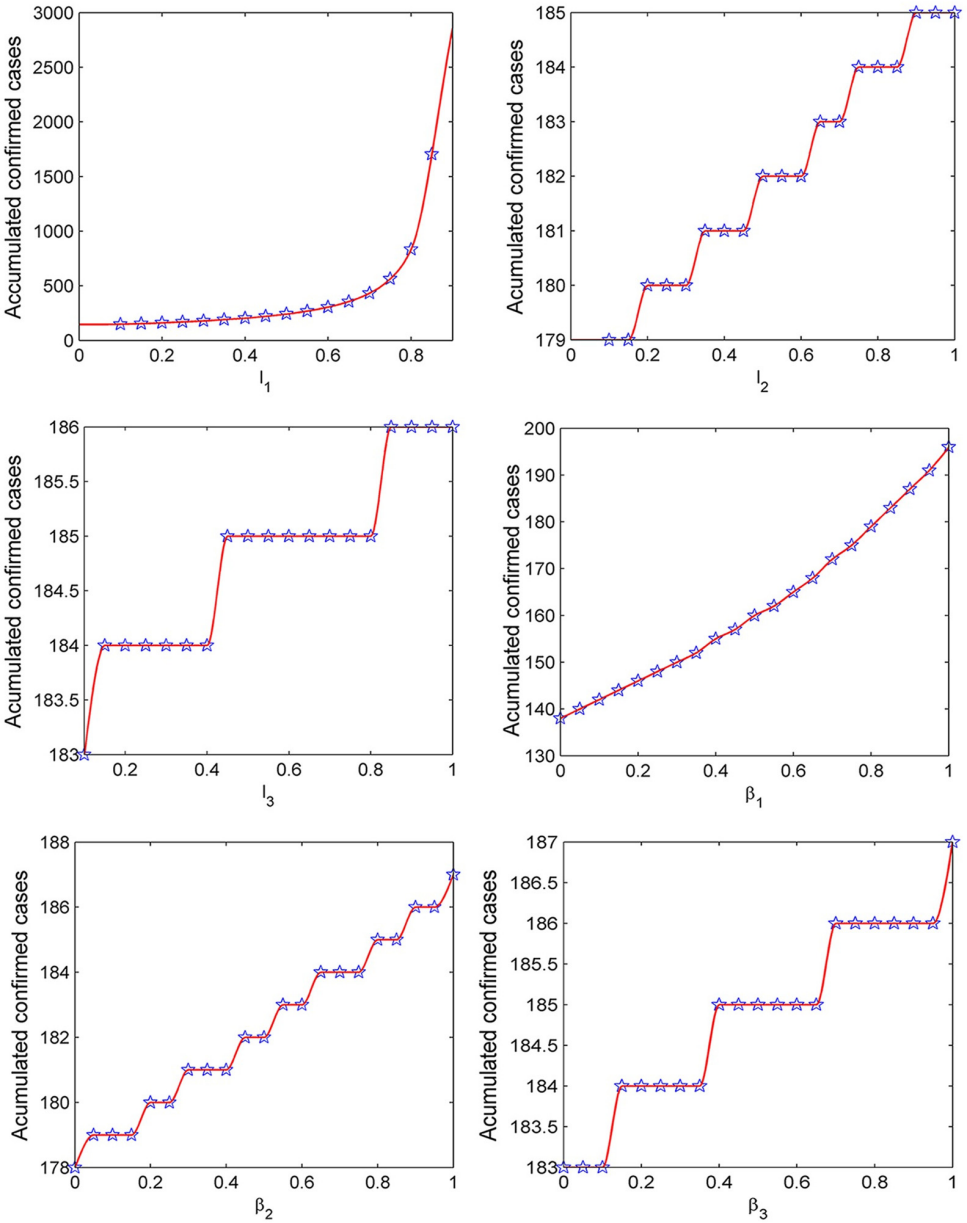
The relationships between total number of confirmed cases and transmission coefficient and self-protection coefficient (here the smaller value of self-protection coefficient, the lower probability of being infected).

From Fig 7, it is obvious that the bigger value the self-protective coefficient, the smaller the final size of accumulated confirmed cases. Moreover, the bigger the transmission coefficient, the bigger size the final size of accumulated infected cases. The parameters *l*_1_ and *β*_1_ have a bigger influence on the final size of accumulated confirmed cases, and this is consistent with the sensitivity analysis results of *R*_0_. As a result, it is critical to isolate asymptomatic cases. Through calculating, we get the theoretical expression of final accumulated confirmed cases size which is shown in S1 File.

## Discussion

Combining with the actual propagation situation and patients’ data from May 20 to July 4, 2015 in the Republic of Korea, we establish two dynamical Models (1) and (3) study the process of MERS in the Republic of Korea. When the novel disease emerged, there were no effective control measures for many reasons, in this case the basic reproduction number *R*_0_ = 4.42, i.e., the epidemic spread quickly. Since South Korean Government isolated or monitored all the close contacts with the patients infected by MERS-CoV, the additional confirmed cases gradually reduced and epidemic was controlled quickly. At this moment, *R*_*c*_ = 0.385. After July 5, there is no additional confirmed cases reported anymore.

Applying our Models (1) and (3), it comes to a consistent conclusion through the sensitivity of the basic reproduction and the final size of accumulated confirmed cases: isolating all the close contacts and strengthening the self-protection ability of susceptible are the most effective control measures.

During this period, the spread situation of MERS in China also confirmed the above results. In May 29, 2015, the first confirmed case in China was reported in Huizhou, Guongdong province [33]. The patient is a Korean in his mid-40s, who traveled to Guangdong province, China via Hong Kong on May 26. Subsequently, the China Government isolated all close contacts as soon as possible and publicized the progress of the event to the society, killed the source of disease in the bud. Therefore, MERS cannot spread across population under proper control measures.

Based on the successful experience in control MERS, we give some specific suggestions in face with emerging diseases: ①: Limit or stop taking fairs, rallies and theaters performances; ②: Provide necessary personal protective equipment to staff participated in emergency response; ③: Timely publish the information of emergence disease to the community, including the number confirmed cases, suspected cases, hospitalized cases and so on; ④: Carry out targeted health care education, raise public awareness and slef-protection and eliminate public mental disorder.

The MERS mainly occurs in the Middle East countries, where the epidemic is most severe in Saudi Arabia. There was a large-scale outbreak from early April to late June in Saudi Arabia in 2014, while the outbreak in the Republic of Korea mainly emerged from mid-May to early July in 2015. Why the outbreaks of MERS are centralized from April to July and whether the outbreak of MERS has relation with season [34] are needed to be checked by more investigations. In addition, it is also a good method in studying infectious disease on complex networks [35–37] and we will try to study disease-behavior dynamics on complex networks in the future study.

## Supporting Information

S1 File Mathematical analysis. (PDF)
