# Supplementary material for: Modeling the Transmission of Middle East Respirator Syndrome Corona Virus in the Republic of Korea
Source: PLoS One. 2015 Dec 21;10(12):e0144778. doi: 10.1371/journal.pone.0144778 (PMC4686901; doi:10.1371/journal.pone.0144778)
Supplement: S1 File — (PDF) [file pone.0144778.s001.pdf]

Modeling the Transmission of Middle East Respirator Syndrome Corona virus in Republic of Korea

Zhi-Qiang Xia[1,2], Juan Zhang[2], Ya-Kui Xue[1], Gui-Quan Sun[2], Zhen Jin[2]
1 Department of Mathematics, North University of China, Taiyuan, Shanxi 030051, PR China
2 Complex Systems Research Center, Shanxi University, Taiyuan, Shanxi 030051, PR China

S1 File. Mathematical analysis.

A. The basic reproduction number R_0 of system (1)

Through simple calculation, we can obtain a disease free equilibrium $E_1 = (S_1, 0, 0, 0, 0, R_1)$. Then we use the spectral radius of the next generation matrix (the method proposed by the Van Den Driessche in [1]) to get the expression of R_0 of system (1) . Firstly, only the infected compartments satisfied by the following system are considered:

$$\begin{cases} \frac{dE}{dt} = \beta_1 \frac{SA}{N} + \beta_2 \frac{SI}{N} + \beta_3 \frac{SH}{N} - \sigma E, \\ \frac{dA}{dt} = (1-\gamma)\sigma E - k_1 A, \\ \frac{dI}{dt} = \gamma\sigma E - \lambda I, \\ \frac{dH}{dt} = \lambda I - k_2 H - \delta H. \end{cases}$$

And,

$$\mathcal{F} = \begin{pmatrix} \beta_1 \frac{S_0 A}{N} + \beta_2 \frac{S_0 I}{N} + \beta_3 \frac{S_0 H}{N} \\ 0 \\ 0 \\ 0 \end{pmatrix}, \qquad \mathcal{V} = \begin{pmatrix} \sigma E \\ k_1 A - (1-\gamma)\sigma E \\ \lambda I - \gamma\sigma E \\ k_2 H + \delta H - \lambda I \end{pmatrix},$$

where $\mathcal{F}$ denotes the rate of appearance of new infection and $\mathcal{V}$ denotes the rate of transfer of individuals. Calculating the derivative of $\mathcal{F}$ and $\mathcal{V}$ about $x = (E, A, I, H)$, respectively. Then substitute initial data$(S_0, E_0, A_0, I_0, H_0, R_0)$ into variables, we can get:

$$F = \begin{pmatrix} 0 & \beta_1 \frac{S_0}{N} & \beta_2 \frac{S_0}{N} & \beta_3 \frac{S_0}{N} \\ 0 & 0 & 0 & 0 \\ 0 & 0 & 0 & 0 \\ 0 & 0 & 0 & 0 \end{pmatrix}, \qquad V = \begin{pmatrix} \sigma & 0 & 0 & 0 \\ -(1-\gamma)\sigma & k_1 & 0 & 0 \\ -\sigma\gamma & 0 & \lambda & 0 \\ 0 & 0 & -\lambda & \delta + k_2 \end{pmatrix},$$

and thus

$$FV^{-1} = \begin{pmatrix} \frac{(1-\gamma)\beta_1 S_0}{k_1 N} + \frac{\gamma\beta_2 S_0}{\lambda N} + \frac{\gamma\beta_3 S_0}{(k_2+\delta)N} & \frac{\beta_1 S_0}{k_1 N} & \frac{\beta_2 S_0}{\lambda N} + \frac{\beta_3 S_0}{\delta + k_2} & \frac{\beta_3 S_0}{(\delta_2 + k_2)N} \\ 0 & 0 & 0 & 0 \\ 0 & 0 & 0 & 0 \\ 0 & 0 & 0 & 0 \end{pmatrix},$$

where R_0 is the biggest eigenvalue of matrix FV^{-1} i.e. $R_0 = \rho(FV^{-1})$. The expression of R_0 is as follows:

$$R_0 = \frac{(1-\gamma)\beta_1 S_0}{k_1 N} + \frac{\gamma\beta_2 S_0}{\lambda N} + \frac{\gamma\beta_3 S_0}{(k_2+\delta)N}.$$

B. The basic reproduction number R_c of system (3)

In this part, we give the expression of R_c:

$$R_c = \frac{(1-\gamma)l_1\beta_1 S^0}{(d_1+\sigma)(d_2+k_1)N} + \frac{\gamma\sigma l_2\beta_2 S^0}{(d_1+\sigma)(d_3+\lambda)N} + \frac{\gamma\lambda\sigma l_3\beta_3 S^0}{(d_1+\sigma)(d_3+\lambda)(d_4+k_2+\delta)N}.$$

C. The Final Size Relation

The actual situation is that there is no additional confirmed cases reported from July 4, 2015. In this case we are more concerned about the final size of confirmed cases during disease prevalence process [2], when the disease of MERS has already die out, i.e., $E(+\infty)=0$, $A(+\infty)=0$, $I(+\infty)=0$, $H(+\infty)=0$. Note that all solutions of model (3) remain non-negative and bounded in the set defined by $S(t), E(t), A(t), I(t), H(t), R(t) \geq 0$. It is easy to obtain:

$$\frac{d}{dt}[S(t)+E(t)] = -(\sigma+d_1)E.$$

It is found that $S(t)+E(t)$ is decreasing while $E(t)>0$. Lower Bound of $S(t)+E(t)$ is 0. However, $E(+\infty)=0$, so $\lim_{t\to+\infty}(S^{'}(t)+E^{'}(t))=0$. For an arbitrary function $x(t)$ with non-negative components, $x_{+\infty}=\lim_{t\to+\infty} x(t)$ and $\overline{x}=\int_0^{+\infty} x(t)dt$. Apply this equation, we integrate the above equation from $t=0$ to $+\infty$:

$$E(0)-E(+\infty)+S(0)-S(+\infty) = (\sigma+d_1)\int_0^{+\infty} E(t)dt = (\sigma+d_1)\overline{E}, \ \ and \ \ E(+\infty)=0$$

i.e.,

$$\overline{E} = \frac{S(0)+E(0)-S(+\infty)}{\sigma+d_1}.$$

Similarly, we have:

$$A(+\infty)-A(0) = (1-\gamma)\sigma\overline{E} - (k_1+d_2)\overline{A}, \ \ and \ \ A(+\infty)=0$$

$$\overline{A} = \frac{A(0)+(1-\gamma)\sigma\overline{E}}{k_1+d_2}.$$

$$I(+\infty)-I(0) = \gamma\sigma\overline{E} - (\lambda+d_3)\overline{I}, \ \ and \ \ I(+\infty)=0$$

$$\overline{I} = \frac{I(0)+\gamma\sigma\overline{E}}{\lambda+d_3}.$$

$$H(+\infty)-H(0) = \lambda\overline{I} - (k_2+\delta+d_4)\overline{H}, \ \ and \ \ H(+\infty)=0$$

$$\overline{H} = \frac{H(0)+\lambda\overline{I}}{k_2+\delta+d_4}.$$

It is easy to get:

$$\overline{A} = \frac{A(0)+(1-\gamma)\sigma\overline{E}}{k_1+d_2} = \frac{(\sigma+d_1)A(0)+\sigma(1-\gamma)[S(0)+E(0)-S(+\infty)]}{(\sigma+d_1)(k_1+d_2)}.$$

$$\overline{I} = \frac{I(0) + \gamma\sigma\overline{E}}{\lambda + d_3} = \frac{(\sigma + d_1)I(0) + \gamma\sigma[S(0) + E(0) - S(+\infty)]}{(\sigma + d_1)(\lambda + d_3)}.$$

$$\overline{H} = \frac{H(0) + \lambda\overline{I}}{k_2 + \delta + d_4} = \frac{(\sigma + d_1)(\lambda + d_3)H(0) + \lambda(\sigma + d_1)I(0) + \lambda\gamma\sigma[S(0) + E(0) - S(+\infty)]}{(\sigma + d_1)(\lambda + d_3)(k_2 + \delta + d_4)}.$$

Integration of the first equation of model(3) from 0 to t_1, we get:

$$\ln\frac{S(0)}{S(t_1)} = \int_0^{t_1}[\frac{l_1\beta_1 A(t) + l_2\beta_2 I(t) + l_3\beta_3 H(t)}{N}]dt.$$

Letting $t_1 \to +\infty$, we have:

$$\begin{aligned}\ln\frac{S(0)}{S(+\infty)} = & \frac{1}{N}\{\frac{(\sigma + d_1)A(0) + \sigma(1-\gamma)[S(0) + E(0) - S(+\infty)]}{(\sigma + d_1)(k_1 + d_2)}l_1\beta_1 \\ & + \frac{(\sigma + d_1)I(0) + \gamma\sigma[S(0) + E(0) - S(+\infty)]}{(\sigma + d_1)(\lambda + d_3)}l_2\beta_2 \\ & + \frac{(\sigma + d_1)(\lambda + d_3)H(0) + \lambda(\sigma + d_1)I(0) + \lambda\gamma\sigma[S(0) + E(0) - S(+\infty)]}{(\sigma + d_1)(\lambda + d_3)(k_2 + \delta + d_4)}l_3\beta_3\}.\end{aligned}$$

Since $S(0)$, $A(0)$, $E(0)$, $I(0)$ and $H(0)$ are known, we obtain a relation about the final size of accumulated confirmed cases.

References

1. Van den Driessche P, Watmough J (2002) Reproduction numbers and sub-threshold endemic equilibria for compartmental models of disease transmission. *Mathematical biosciences* 180: 29-48

2. Jin Z, Zhang J, Song L P, et al. (2011) Modelling and analysis of influenza A (H1N1) on networks. *BMC public health* Suppl 1: S9.
